# Topical larynx lidocaine Spraying reduces cardiovascular stress response caused by suspension laryngoscopic surgery

**DOI:** 10.1016/j.bjorl.2024.101481

**Published:** 2024-08-05

**Authors:** Liu Huan, Bu Wenhao, Chen Xiangdong, Wu Zhilin

**Affiliations:** a Huazhong University of Science and Technology, Tongji Medical College, Union Hospital, Department of Anesthesiology, Wuhan, China; b Huazhong University of Science and Technology, Tongji Medical College, Union Hospital, Institute of Anesthesia and Critical Care Medicine, Wuhan, China; c Key Laboratory of Anesthesiology and Resuscitation (Huazhong University of Science and Technology), Ministry of Education, China; d Maternal and Child Health Hospital of Hubei Province, Department of Anesthesiology, Wuhan, China

**Keywords:** Lidocaine, Laryngoscopic, Hemodynamics, Epinephrine, Norepinephrine

## Abstract

**Objective:**

This study aimed to investigate the potential reduction of cardiovascular stress response caused by suspension laryngoscopic surgery through the application of lidocaine spray on the larynx and trachea.

**Methods:**

A total of 68 patients scheduled for elective suspension laryngoscopic surgery were randomly assigned to either the lidocaine group (Group L, n = 34) or the control group (Group C, n = 33). In Group L, patients received a sprayed lidocaine dose of 2 mg/kg on the larynx and trachea after anesthesia induction, prior to intubation. In Group C, equal volumes of saline solution were administered. MAP and HR were recorded at various time points: before anesthesia (T0), 1-minute after intubation (T1), 1 and 3 min after suspension laryngoscopy (T2 and T3), at the end of the operation (T4), and at 1, 5, and 30 min after extubation (T5, T6, and T7). Arterial blood glucose, epinephrine, and norepinephrine levels were measured at T0, T2, T5, and T7. The occurrence of severe cough and sore throat at T6 and T7 after extubation was compared between the two groups.

**Results:**

At T0 and T1, there were no statistically significant differences in mean arterial pressures, heart rate, and blood catecholamine levels between the two groups. However, from T2 to T7, the blood pressure and heart rate in Group L were lower compared to Group C, with significant differences observed at T2‒T6 (*p* < 0.05). Group L also showed less elevation in blood glucose at T2, T5, and T7 (*p* < 0.05). The changes in epinephrine and norepinephrine levels between the two groups were statistically significant at T2 and T5 (*p* < 0.05).

**Conclusions:**

Administering lidocaine spray on the larynx and trachea during intubation for suspension laryngoscopic surgery can effectively alleviate the stress response.

**Level 1 evidence:**

Patients in this study are randomly assigned to the treatment or control group and are followed prospectively.

## Highlights

• Suspension laryngoscopic surgery leads to cardiovascular stress response.
• Sprayed lidocaine on the surgical area can reduce the stress response.
• Adrenaline and norepinephrine were less elevated in lidocaine spraying group.

## Introduction

Suspension laryngoscopic surgery, a commonly performed short-term operation under general anesthesia,^1^ often leads to a pronounced cardiovascular stress response.^2^ This response is primarily triggered by the stronger and more enduring stimulation from the suspension laryngoscope and surgical procedures compared to tracheal intubation.^3^ The resulting excitation of the superior laryngeal nerve and sympathetic nerve leads to the release of large amounts of catecholamines, subsequently causing elevated blood pressure, rapid heart rate, and an increased risk of cardiovascular complications. This poses a significant threat to patients, particularly those with cardiovascular and cerebrovascular diseases.^4^ It also produces postoperative adverse reactions, such as cough and sore throat in the majority of patients, which might increase the consumption of analgesics and narcotics.^5^

To mitigate the stress response and reduce postoperative adverse reactions in suspension laryngoscopic surgery, various approaches have been explored in clinical practice. These include deepening anesthesia,^6^ blocking the internal branch of the superior laryngeal nerve (SLN),^7^ and intravenous lidocaine administration.^8^ Additionally, a research has shown that the application of sprayed lidocaine on the surgical area after suspension laryngoscopy can effectively inhibit the increase in both heart rate and blood pressure, meanwhile, reduce the coughing reflex before or during arousal.^9^

However, existing research on lidocaine's inhibitory effects on stress reactions has primarily focused on monitoring changes in patients' vital signs, with limited investigation into alterations in blood catecholamine levels. In this study, we aimed to evaluate the impact of lidocaine spray on the trachea and throat during intubation on stress response during suspension laryngoscopic surgery. Specifically, we recorded mean arterial blood pressure, heart rate, blood glucose, adrenaline, and norepinephrine levels at various time points after lidocaine spraying. Additionally, we monitored the occurrence of severe cough and sore throat following extubation. These findings will contribute valuable insights for the clinical application of lidocaine in managing stress response during suspension laryngoscopic surgery.

## Methods

### General information

This study was conducted with the approval of the Ethics Committee of Maternal and Child Health Hospital of Hubei Province, and all participants provided written informed consent. Patients who underwent suspension laryngoscopic surgery in our hospital from May 2023 to October 2023 were included in the study. Using a random number table, patients were allocated into either the lidocaine group (group L, n = 34) or the control group (group C, n = 33). The saline and lidocaine solutions were prepared by an anesthesiologist who was not involved in the study. All participants, including attending anaesthetists, operating otolaryngologist and investigators involved in data collection were unaware of the group allocation. After the completion of the study, the codes were broken down. Inclusion criteria included patients diagnosed with benign laryngeal lesions (cysts/polyps/nodules, etc.) and scheduled for suspension laryngoscopic surgery, aged between 18 and 65 years with ASA (American Society of Anesthesiologists^10^ physical status of I–II, Exclusion criteria included difficult airway, severe allergies to lidocaine, serious respiratory and cardiovascular diseases, psychiatric disorders, and use of analgesics before surgery.

### Procedures

The anesthesia procedure was conducted by anesthesiologist who was unaware of the group assignments. Radial artery puncture was performed under local anesthesia in both groups. Continuous monitoring of electrocardiogram (ECG), heart rate (HR), invasive arterial blood pressure (ART), and pulse oxygen saturation (SpO 2) was carried out and recorded automatically every 5 min. Vital signs were recorded as baseline (T0) prior to induction of anesthesia. Anesthesia was induced with 2.5 mg/kg propofol and 0.3 μg/kg sufentanil, followed by 0.2 mg/kg cisatracurium as muscle relaxant. Orotracheal intubation was performed after ventilation with 100% oxygen via a face-mask for 3 min. Lidocaine (2 mg/kg) was sprayed on the larynx and trachea during intratracheal intubation, followed by intubation. Patients in group C received equal volumes of saline solution. Maintenance of anesthesia was achieved with propofol 4−6 mg/kg/h and remifentanil 0.2−0.4 ug/kg/min. If HR and ART changed more than 20% of baseline value, 10ug of remifentanil was administered intravenously. The laryngeal microsurgery was performed by an experienced ENT surgeon using ablation electrode to remove the lesion and coagulate the bleeding under laryngoscope. After surgery, patients were transferred to the post anesthesia care unit, and 1 mg of neostigmine was administered intravenously to antagonize cisatracurium before arousal. Tracheal extubation was completed after observing full recovery with the ability to follow verbal commands. Operative time was defined as the time between implantation and removal of the suspension laryngoscope during surgery. Extubation time was recorded as the time from drug withdrawal after operation to extubation.

### Clinical observations

The primary outcome measures included Mean Arterial Blood Pressure (MAP) and levels of catecholamine. MAP was recorded at various time points: before anesthesia (T0), 1 min after intubation (T1), 1 and 3 min after suspension laryngoscopy (T2 and T3), at the end of the operation (T4), and at 1, 5, and 30 min after extubation (T5, T6, and T7 respectively). Arterial blood glucose and levels of catecholamine (epinephrine and norepinephrine) were measured at T0, T2, T5, and T7. Additionally, the occurrence of severe cough and sore throat after extubation was recorded at T6 and T7. Severe postoperative sore throat was defined as complaints of sore throat or severe pain associated with a noticeable change in voice, while severe postoperative cough was defined as more than one episode of unsustained (65 s) cough or sustained (65 s) and repetitive cough with head lift.^11^

### Sample size and statistical analysis

Based on the literature, the baseline value of plasma noradrenaline concentrations was 2.09 ± 0.74 nmol/L.^12^ Laryngoscopy and tracheal intubation can cause a 34–74% increase in plasma concentrations.^12^ We hypothesize that topical larynx lidocaine Spraying could reduce the noradrenaline elevation by 20 percent, so the noradrenaline concentrations in group L were approximately 0.6 nmol/L lower than those in group C. Using a two-sided alpha of 0.05 and 90% power, as described by Wang et al.,^13^ the calculated sample size was 32 cases in each group. Accounting for a dropout rate of 20% in each group, 40 cases were included in each group.

Data analysis was performed using SPSS22.0 statistical software. The Kolmogorov-Smirnov test was used to check for normal distribution. Normally distributed continuous variables were presented as mean ± standard deviation (mean ± SD) and analyzed using the independent Student's t-test. Non-normally distributed variables were presented as the median and interquartile range (IQR), compared using the Mann–Whitney U test. Enumeration variables were presented as count (percentage) and compared with Chi-Square test and Fisher’s exact test. *p* < 0.05 was considered as signiﬁcant.

## Results

**Fig. 1:**
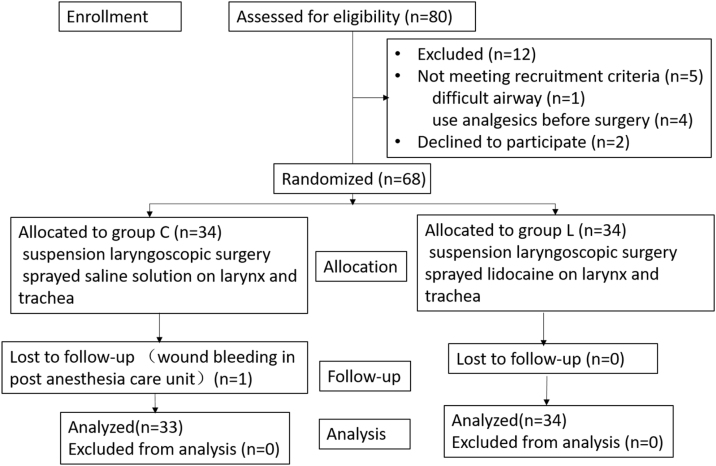
Consolidated standards of reporting trials (CONSORT) flow diagram of patients’ distribution.

From May to October 2023, a total of 80 patients undergoing elective suspension laryngoscopic surgery were initially screened. Twelve patients were excluded due to not meeting the recruitment criteria, refusal to participate, change in the type of surgery, or declining to participate. Ultimately, 68 patients were enrolled and randomly assigned to either Group C (n = 34) or Group L (n = 34). One patient from Group C was lost to follow-up due to post-anesthesia care unit wound bleeding, resulting in reoperation. Consequently, all 33 subjects in Group C and 34 subjects in Group L were included in the result analysis (Fig. 1).

There were no significant differences in age, ASA grade, sex distribution, operative time, extubation time and propofol consumption between the two groups (*p* > 0.05). The doses of remifentanil was less in Group L (*p* < 0.05) (refer to Table 1).

**Table 1:** Basic participant characteristics according to study group.

Characteristic	Group C (n = 33)	Group L (n = 34)	*p* value
Age(year)	45 (36−56)	44 (33−53)	0.38
Sex (male/female)	17 (51.5%)/16 (48.5%)	19 (55.9%)/15 (44.1%)	0.72
ASA I/II	23 (69.7%)/10 (30.3%)	26 (76.5%)/8 (23.5%)	0.53
BMI, kg/m^2^	24.0 ± 3.29	24.21 ± 3.03	0.79
operative time (min)	19.76 ± 4.52	20.47 ± 5.16	0.55
extubation time (min)	15.61 ± 3.79	14.03 ± 3.04	0.06
Propofol total dose (mg)	307.88 ± 51.62	318.97 ± 50.62	0.38
Remifentanil total dose (ug)	362.27 ± 61.91	333.24 ± 45.97	0.03

Group C, Control group; Group L, Lidocaine group. The values are mean ± SD or median (Q1-Q3) or number of patients (%). ASA, American Society of Anesthesiologists; BMI, Body Mass Index.

There were no statistically significant differences in blood pressure, heart rate, blood glucose, and catecholamine (norepinephrine and epinephrine) levels between the two groups at the T0 and T1 time points. However, the blood pressure and heart rate of both groups at the T2‒T7 time points were higher than those at T0. Compared to Group C, Group L exhibited lower blood pressure and heart rate, with statistically significant differences observed at time points T2‒T6 (*p* < 0.05). Furthermore, blood glucose levels at T2, T5, and T7 were less elevated in Group L compared to Group C, while the levels of epinephrine and norepinephrine were significantly less elevated at T2 and T5 in Group L (*p* < 0.05) (Table 2).

**Table 2:** Comparison of MAP, HR, Glu, NE an E between the two groups at specified time points.

		T0	T1	T2	T3	T4	T5	T6	T7
MAP (mmHg)	Group C	93.03 ± 9.92	103.58 ± 7.59	106.12 ± 8.21	100.45 + 6.27	96.30 ± 7.23	102.76 ± 7.47	99.64 + 6.72	94.61 ± 9.36
	Group L	93.79 ± 10.01	104.65 ± 7.32	102.09 ± 5.91	96.79 ± 6.14	92.64 ± 7.03	98.88 ± 6.51	96.06 ± 5.86	91.12 ± 10.54
	*p* value	0.75	0.56	0.02	0.02	0.04	0.03	0.02	0.15
HR (bpm)	Group C	76.48 ± 12.26	90.12 ± 9.35	93.85 ± 8.35	88.52 ± 8.44	79.27 ± 10.75	92.69 ± 9.49	85.30 ± 8.91	75.45 ± 9.43
	Group L	74.97 ± 10.85	91.55 ± 10.00	84.18 ± 8.68	79.76 ± 7.57	74.29 ± 8.89	86.26 ± 8.84	79.94 ± 8.08	72.74 ± 8.28
	*p* value	0.59	0.55	＜0.01	＜0.01	0.04	＜0.01	0.01	0.21
Glu (mmol/L)	Group C	5.38 ± 0.72		5.78 ± 0.68			6.19 ± 0.25		6.34 ± 0.87
	Group L	5.27 ± 0.59		5.46.±0.55			5.77 ± 0.48		5.81 ± 0.52
	*p* value	0.50		0.04			＜0.01		＜0.01
NE (nmol/L)	Group C	1.95 ± 0.29		2.61 ± 0.59			2.67 ± 0.57		2.27 ± 0.34
	Group L	1.96 ± 0.27		2.33 ± 0.29			2.39 ± 0.28		2.18 ± 0.28
	*p* value	0.84		0.02			0.01		0.27
E (nmol/L)	Group C	1.89 ± 0.25		2.45 ± 0.43			2.51 ± 0.52		2.20 ± 0.33
	Group L	1.91 ± 0.29		2.24 ± 0.27			2.30 ± 0.23		2.07 ± 0.25
	*p* value	0.79		0.02			0.03		0.07

Group C, Control group; Group L, Lidocaine group. Values are mean ± SD. MAP, Arterial Pressure; HR, Heart rate min^−1^; Glu, Blood glucose; NE, Norepinephrine; E, Adrenaline.

The incidence of severe cough was significantly lower in Group L than in Group C at T6 (5 min after extubation). 12 patients had severe cough in control group and 4 cases in Group L at T7. However, this difference was not statistically significant. Similarly, the incidence of severe sore throat following extubation in Group L was significantly lower than that in Group C at T6 (5 min after extubation) and T7 (30 min after extubation), with statistically significant differences observed (*p* < 0.05) (Table 3).

**Table 3:** Comparison of severe POST and cough between the two groups at specified time points.

		T6	T7
Severe cough	Group C	18(54.5%)	12(0.3%)
	Group L	7(5.6%)	4(0.3%)
	*p* value	<0.01	0.18
Severe POST	Group C	30(33.3%)	20(25%)
	Group L	5(13.9%)	3(5.6%)
	*p* value	<0.01	<0.01

Group C, Control group; Group L, Lidocaine group. The values indicate number of patients (%).

## Discussion

This study demonstrated that the administration of lidocaine spray on the larynx and trachea during intubation could not only mitigate cardiovascular stress response, but also reduce postoperative complications, such as severe sore throat and cough, associated with suspension laryngoscopic surgery.

Endotracheal intubation during this surgery can cause the excitation of superior laryngeal nerve and laryngeal sympathetic nerve, resulting in hemodynamic changes.^14^ A study confirmed that IV or topical administration of lignocaine was effective in attenuating the haemodynamic responses to endotracheal intubation during the induction of anaesthesia.^15^ However, in our study, we choose to spray lidocaine on the larynx and trachea during intubation but not in advance for one reason. Lidocaine spray during the anesthesia induction can minimize the discomfort of awake lidocaine application. And an adequate depth of anesthesia prior to intubation can minimize the risk of unstable hemodynamics.^16^ The lidocaine dosage (2 mg/kg) in this study is based on a previous research that demonstrated its benefits without toxic symptoms.^17^

The suspension laryngoscopic surgery induces intense and persistent excitation of the superior laryngeal and sympathetic nerves, leading to potentially life-threatening cardiovascular responses.^18^ Previous studies have shown that spraying lidocaine on the larynx and upper airway can reduce the systemic sympathetic reflex during suspension laryngoscopic surgery and maintain stable hemodynamics.^15^, ^19^ Our study confirmed these findings by observing a smaller increase in blood pressure and heart rate in the lidocaine group compared to the control group after laryngoscope implantation and extubation. Additionally, it took more than 2 min for lidocaine to reach the effective concentration in our study, which explains the lack of significant differences in mean blood pressure and heart rate between the two groups 1 min after intubation.

Laryngoscope-induced stress response results in elevated plasma catecholamine concentrations and blood glucose levels.^20^ Changes in plasma noradrenaline and adrenaline and arterial blood pressure have been shown to be similar.^21^ Plasma noradrenaline concentrations have increased 34–74% after laryngoscopy and tracheal intubation.^12^ The use of local anesthetics such as aerosolized lidocaine will reduce the effect of device stimulation, but it is more effective to reduce the stimulation itself.^22^ In our experiment, we hypothesized that spraying lidocaine would block the superior laryngeal nerve and laryngeal sympathetic nerve, thereby partially alleviating the stress response caused by suspension laryngoscopic surgery. To investigate this hypothesis, we measured blood glucose, epinephrine, and norepinephrine levels in arterial blood at different time points after endotracheal intubation, laryngoscope implantation, and extubation. The results revealed that the increase in plasma concentrations of blood glucose, epinephrine, and norepinephrine in the lidocaine group was not as significant as in the control group after the implantation of the suspension laryngoscope and during the extubation period. This further supports the notion that lidocaine spray during intubation can effectively reduce the stress response associated with suspension laryngoscopic surgery.^23^ When blood pressure increased by 20%, we adopted deepening anesthesia as a method to manage the hemodynamic changes.^6^ The dose of remifentanil consumption in group L was less, demonstrating the analgesic effect of lidocaine spraying indirectly. However, it is worth noting that deepening anesthesia may prolong patient recovery time,^24^ no statistical difference was observed in our study, possibly because the sample size was not large enough.

Sore throat and cough are common complications after suspension laryngoscopy.^25^ Mostly, the symptoms of sore throat subside within 6 h and fully recover within 24 h after surgery, they are considered preventable side effects.^26^ These complications can be attributed to airway insults resulting from endotracheal intubation and extubation under general anesthesia. However, suspension laryngoscopy can irritate the superior laryngeal nerve and sympathetic nerve, leading to prolonged local and systemic effects and exacerbating cough and sore throat after extubation, thereby intensifying the stress response. However, the application of lidocaine spray on the larynx and trachea provides specific local anesthesia, effectively inhibiting these reflexes. Our study confirmed that lidocaine spray on the larynx and trachea can alleviate severe sore throat and cough after surgery, which is consistent with the finding of previous study.^9^

There are several limitations in our study. First, we only include patients with ASA I–II, benefits from lidocaine spay in suspension laryngoscopy in higher ASA grade patients is to be confirmed. Second, the sample size in this study is relatively small. And only lidocaine was tested in the experiment, and its effect was evaluated only for the 30-minute post extubation. Studies with a larger sample size, different types of local anesthetics, and longer follow-up for benefits evaluation are to be carried out.

## Conclusion

Suspension laryngoscopy surgery elicits a cardiovascular response through the activation of the sympathetic nervous system. The administration of lidocaine spray on the larynx and trachea has been demonstrated to mitigate the stress response, stabilize blood pressure, decrease catecholamine release, enhance hemodynamic stability, and reduce the incidence of postoperative complications such as severe sore throat and cough. This approach is effective and easily to perform, and is worthy of widely application in suspension laryngoscopy surgery.

## Funding

The authors received no funding for this work.

## Conflicts of interest

The authors declare no conflicts of interest.

## References

[1] Hunsaker D.H. (1994). Anesthesia for microlaryngeal surgery: the case for subglottic jet ventilation. Laryngoscope.

[2] Lakhe G., Pradhan S., Dhakal S. (2021). Hemodynamic response to laryngoscopy andintubation using McCoy laryngoscope: a descriptive cross-sectional study. JNMA J Nepal Med Assoc..

[3] Strong M.S., Vaughan C.W., Mahler D.L., Jaffe D.R., Sullivan R.C. (1974). Cardiac complications of microsurgery of the larynx: etiology, incidence and prevention. Laryngoscope.

[4] Lakhe G., Pradhan S., Dhakal S. (2021). Hemodynamic response to laryngoscopy and intubation using McCoy laryngoscope: a descriptive cross-sectional study. JNMA J Nepal Med Assoc..

[5] Taliercio S., Sanders B., Achlatis S., Fang Y., Branski R., Amin M. (2017). Factors associated with the use of postoperative analgesics in patients undergoing direct microlaryngoscopy. Ann Otol Rhinol Laryngol..

[6] Lin Z.J., Chen X.F., Zhou M. (2021). Effect of anesthesia induction with lidocaine combined with remifentanil on the response to tracheal intubation in patients with hypertension. Chinese Foreign Med Res..

[7] Bao Y., Xiong J., Wang H., Zhang Y., Zhong Q., Wang G. (2022). Ultrasound-guided block ofthe internal branch of the superior laryngeal nerve reduces postoperative sore throat caused by suspension laryngoscopic surgery: a prospective randomized trial. Front Surg..

[8] Abou-Madi M.N., Keszler H., Yacoub J.M. (1977). Cardiovascular reactions to laryngoscopy and tracheal intubation following small and large intravenous doses of lidocaine. Can Anaesth Soc J..

[9] Paltura C., Güvenç A., Develioglu ÖN., Yelken K., Külekçi M. (2020). Original research: aerosolized lidocaine: effective for safer arousal after suspension laryngoscopy. J Voice..

[10] Haynes S.R., Lawler P.G. (1995). An assessment of the consistency of ASA physical status classification allocation. Anaesthesia..

[11] Ramkumar R., Arora S., Bhatia N., Bansal S. (2019). Ultrasound guided superior laryngeal nerve block as an adjuvant to general anesthesia during endoscopic laryngeal surgery: a prospective, randomized, double-blind trial. Am J Otolaryngol..

[12] McCoy E.P., Mirakhur R.K., McCloskey B.V. (1995). A comparison of the stress response to laryngoscopy. The Macintosh versus the McCoy blade. Anaesthesia..

[13] Wang X., Ji X. (2020). Sample size estimation in clinical research: from randomized controlled trials to observational studies. Chest..

[14] Capuzzo M., Verri M., Alvisi R. (2010). Hemodynamic responses to laryngoscopy and intubation: etiological or symptomatic prevention?. Minerva Anestesiol..

[15] Kocamanoglu I.S., Cengel Kurnaz S., Tur A. (2015). Effects of lignocaine on pressor response to laryngoscopy and endotracheal intubation during general anaesthesia in rigid suspension laryngoscopy. J Laryngol Otol..

[16] Kim J.T., Shim J.K., Kim S.H., Ryu H.G., Yoon S.Z., Jeon Y.S. (2007). Remifentanil vs. lignocaine for attenuating the haemodynamic response during rapid sequence inductionusing propofol: double-blind randomised clinical trial. Anaesth Intensive Care..

[17] Park Y.O., Bang K.S., Choi E.M., Hong S.J., Kim I.S., Yeong J.Y. (2005). Plasma lignocaine concentration and hemodynamic effect after 10% lignocaine spray on laryngopharyngeal and intratracheal site during the endotracheal intubation. Korean J Anesthesiol..

[18] Justi Cassettari A., Campos E., Santos ÉC., Semenzati G.O., Crespo A.N. (2020). Asystole during suspension laryngoscopy: case report, literature review, and prophylactic strategies. Case Rep Otolaryngol..

[19] Mostafa S.M., Murthy B.V., Barrett P.J., McHugh P. (1999). Comparison of the effects of topical lignocaine spray applied before or after induction of anaesthesia on the pressor response to direct laryngoscopy and intubation. Eur J Anaesthesiol..

[20] Mifsud S., Schembri E.L., Gruppetta M. (2018). Stress-induced hyperglycaemia. Br J Hosp Med (Lond)..

[21] Shribman A.J., Smith G., Achola K.J. (1987). Cardiovascular and catecholamine responses to laryngoscopy with and without tracheal intubation. Br J Anaesth..

[22] Kautto U.M. (1983). Effect of combinations of topical anaesthesia, fentanyl, halothane or N2O on circulatory intubation response in normo- and hypertensive patients. Acta Anaesthesiol Scand..

[23] Lee D.H., Park S.J. (2011). Effects of 10% lidocaine spray on arterial pressure increase due to suspension laryngoscopy and cough during extubation. Korean J Anesthesiol.

[24] Pandazi A.K., Louizos A.A., Davilis D.J., Stivaktakis J.M., Georgiou L.G. (2003). Inhalational anesthetic technique in microlaryngeal surgery: a comparison between sevoflurane-remifentanil and sevoflurane-alfentanil anesthesia. Ann Otol Rhinol Laryngol..

[25] Okui A., Konomi U., Watanabe Y. (2020). Complaints and complications of microlaryngoscopic surgery. J Voice..

[26] Flexman A.M., Duggan L.V. (2019). Postoperative sore throat: inevitable side effect or preventable nuisance? Maux de gorge postopératoires: effet secondaire inéluctable ou désagrément évitable?. Can J Anaesth..

